# The Transactions of the American Medical Association

**Published:** 1856-01

**Authors:** 


ART. VI.— *The Transactions of the American Medical Association. Instituted* 1847. *Vol. VIII.* Philadelphia: Printed for the Association, by T. K. & P. G. Collins. 1855.

A handsomely bound volume, of clear type and good paper, perpetuates the doings of the American Medical Association for the current year. The Committee on Publication have issued it with commendable promptness, and merit the thanks of the Association for the manner in which they have performed their arduous and unrewarded task.

The Proceedings occupy somewhat more space than hitherto, and we but follow the general voice of the profession in saying that they grow in strength as well as size.

Of the business details we need not speak, inasmuch as our report in our June No. was very full. The eloquent and practical address of the President, Dr. Charles A. Pope, of St. Louis, was also reported in full in our pages, so that we have remaining only the various papers read on medical subjects.

The first in order of these is the *Report on the Diseases of Missouri and Iowa.* Thomas Reyburn, M. D., of St. Louis, was the chairman of this committee, and seems to have been the only active member, though he has been materially assisted by local reports from correspondents, and in the meteorological department by Dr. Engleman, of St. Louis. If we are right in assigning so large a modicum of the labor of this document to Dr. Reyburn, we pay no small compliment to his industry and ability as a statician. It is voluminous, comprising 240 pages richly interspersed with extensive tables, together with many more of those condensed tables, the labor of which is so much greater than the uninitiated suppose.

Dr. Reyburn's principal effort seems to have been to embody a mass of facts in tangible form, rather than to educe any theory. This is the more modest course, but the result is none the less profitable. Here is a true history, a collection of the facts and phenomena of disease in a wide spread territory, associated with the topographical and meteorological phenomena in a most masterly manner. To this report the theorist can resort for material to confirm or overturn his labors.

The subject of cholera comes in for that especial notice which its importance demands. In relation to its causation, he remarks that no disease shows more clearly the influence of the "crowd poison" than this. The remark is, we believe, truthful in every locality. Wherever human beings are gathered in crowds we have the cholera tendency manifested. It is in such localities that we have the elements of Dr. Barton's combined causes in greatest force, and while we do not suppose that cholera is destitute of a cause *per se*, we shall attribute to the plain, outside, easily recognizable cause of high temperature and humidity in combination with organic decay, a large share in the propagation of this disease, as of all other zymotics.

We have taken the pains to calculate from Dr. Engleman's reports, the dew point of each of the epidemic months of 1854. We find in

| | | | | | | |
|---|---|---|---|---|---|---|
| May, the Dew Point | | | 56.2°, | Cholera deaths, | | 190 |
| June, " | " | " | 64.3° | " | " | 479 |
| July, " | " | " | 70. ° | " | " | 533 |
| Aug. " | " | " | 67. ° | " | " | 136 |

In these cases the dew point is taken from the mean of the twenty-four hours, which would range from two to five degrees lower than that at 2, P. M. The results are in entire accordance with those elsewhere obtained, and fully support the theory advanced in our paper succeeding Dr. Reyburn's.

We notice that in speaking of "sun-stroke," Dr. R. mentions the occurrence of cases not exposed to the sun, but exposed to great heat and humidity in engine-rooms, and bakeries. Similar cases occurred in New York in 1853, in the laundry of a large hotel, and in sugar refineries. In St. Louis, 22 deaths occurred in the last four days of June. We construct a little table to embody all the facts:

| June. | Deaths by sun-stroke. | Mean Temperature of day. | Mean Temperature of Evaporation. | Highest Temp'ture of Evaporation. |
|---|---|---|---|---|
| 27 | 4 | 86.9 | 76.9 | 79. |
| 28 | 9 | 85.9 | 77.9 | 80.5 |
| 29 | 1 | 86.7 | 77.3 | 80. |
| 30 | 8 | 86.6 | 79.0 | 83. |

During the first five days of July, other deaths by sun-stroke occurred, which we tabulate in the same manner:

| July. | Deaths. | Mean Temperature of the day. | Mean Temperature of Evaporation. | Highest Temp'ture of Evaporation. |
|---|---|---|---|---|
| 1 | 2 | 88.1 | 79.4 | 81.5 |
| 2 | 10 | 86.2 | 78.7 | 81. |
| 3 | 10 | 88.6 | 78.9 | 81.5 |
| 4 | 5 | 87.0 | 79.5 | 82. |
| 5 | 4 | 79.0 | 74.5 | 79. |

The question here arises—what share had heat alone in the causation of these deaths? The average (mean) temperature of the two periods combined (9 days) was 86°. During the remainder of the month of July there were 9 other days, the mean temperature of which exceeded 86°—by 2°. During these latter 9 days only 17 deaths occurred against 53 in the other 9. It is evident, then, that mere temperature is not the condition of cause.

Turning to other conditions, we find no element of difference in the winds, which were southerly on nearly all the days in question. The sky was very clear throughout. It rained on none of these days, and the only point of interest is the fact that on the 30th of June, when 8 deaths occurred by sun-stroke, the day was *cloudy*, the sky being about half covered during the day. This, taken in connection with the in-door deaths, is very good evidence that the direct rays of the sun are not necessary to produce "sun-stroke."

| | | | |
|---|---|---|---|
| The mean temp. of evaporation | for the 9 days having 53 deaths, | was | 78. ° |
| " " " " | " 9 " " 17 " | " | 76.9° |
| " " " " | for the month, | was | 75. ° |

The result here is evident. A temperature of evaporation of 75°, does not seem to be sufficient to cause sun-stroke, except in one instance, that of the 5th of July. On this day 4 deaths occurred under a very cloudy sky, with rain and thunder. The mean temperature was 79° only, while the mean temperature of evaporation was 74.5°, showing a high saturation, the dew point being 73.4°, and the relative humidity 80°. An elevation of 2° from 75°, shows that the fatal point is reached—another degree, added, developes this scourge in its fullest force.

These conclusions are gratifying to us as confirming previous observations. So confident were we that they would confirm them, that we have written this notice of Dr. R.'s report on the supposition that his figures would meet our theory—writing out our theory, first, and *then* making our calculations from Dr. R. to fill the blanks left for figures.

Here we must leave Dr. Reyburn. We have already spent time enough in conning his report, to be assured that it contains a valuable mine for labor, in rearranging the tables for other purposes than the original design. As a whole, Dr. R.'s paper may be justly entitled one of the ablest and most valuable reports yet made to the association—perhaps *the most valuable* of the reports on epidemics.

Following Dr. Reyburn we have the "Report of the Committee on the Hygrometrical Conditions of the Atmosphere in Various Localities, and its Influence on Health."

Our position as author of this report, forbids any formal notice. It has at least one merit—that of brevity—a quality which cost us more labor than would a longer essay.

Following this, again, is the "Report on Deformities after Fractures. By Frank Hastings Hamilton."

Prof. Hamilton has commenced his report in so thorough a manner, that it is not to be expected that he should be able to complete it at once. Accordingly we have in this paper only fractures of the ossa nasi, of the septum narium, of the ossa maxillaria superiora, of the maxilla inferior, and of the clavicle. In so far as the report extends, it is complete. The number of cases analyzed of each of these fractures is, of the ossa nasi, 22; of the septum narium, 7; of the superior maxilla, 6; of the inferior maxilla, 18; and of the clavicle, 53.

These numbers are amply sufficient for analysis, and form undoubtedly the largest collection of cases of fractures ever made *with reference to result.* It is in this light that this report is especially valuable. In all the literature of fracture the idea is conveyed that our rules of management are perfect, and that any resulting deformity is necessarily the fault of the surgeon.

This impression has resulted disastrously to the progress of surgical science, to the reputation of many surgeons, and to the honesty of surgical literature. If all was well, if our present modes of reduction and dressing were the best possible, then where the advantage or utility of further study of the subject, where the necessity of guarding ourselves against suits of malpractice, and where the object in displaying our own ignorance by reporting unfavorable cases?

We rejoice that Prof. Hamilton has had the manliness and courage to speak out on this subject, and become, as it were, the scape-goat of the profession. Ungenerous minds will not be wanting to turn this report to their own advantage, to say that this may be Prof. Hamilton's experience, but not their own, that they (happy men!) find no fault with our existing plans of treatment. Let them beware; for it is not impossible that, in spite of distance or improbability, some of their own failures may be embalmed in the course of the completion of this report!

At the same time the reporter will, we doubt not, recognize the danger of too firm a seat upon a hobby like this—that while he nothing extenuates, neither should he "prove too much"—a result as dangerous to himself as injurious to the profession. The preface of the report assures us that this error is not unseen by its author.

In conclusion let us pay a merited compliment to the neat, sketchy, well-defined, yet unpretentious wood-cuts which illustrate this report.

We defer to our next number the mention of subsequent papers, some of them of great interest.

